# Morphological Study of the Lingula in Adult Human Mandibles of Brazilians Individuals and Clinical Implications

**DOI:** 10.1155/2015/873751

**Published:** 2015-03-02

**Authors:** Nilton Alves, Naira Figueiredo Deana

**Affiliations:** ^1^CIMA Research Group, Faculty of Dentistry, La Frontera University, Francisco Salazar Avenue 1145, P.O. Box 54-D, 4780000 Temuco, Chile; ^2^Passage Frankfurt 1171, Temuco, Chile

## Abstract

*Objectives*. The purpose of this research was to study, in macerated adult human mandibles, the height of the lingula and provide morphometric data for its location considering aspects such as shape of the lingula, gender, and race. *Material and Methods*. 132 macerated mandibles of Brazilian adult individuals, both sexes, Amerindian and Caucasian, were used. The distances: from mandibular notch to lingula; from anterior margin of ramus of mandible to lingula; from posterior margin of ramus of mandible to lingula; from mandibular base to lingula, and the height of lingula were obtained. To perform these measurements we used a digital caliper. The variables such as gender and race were analyzed. *Results*. The mean values found for the height of lingula and its location were determined according to the gender, race, and the lingula shape. *Conclusion*. This research provides additional data on height of the lingula and morphometric data for its location considering aspects such as shape of the lingula, gender, and race, information that had not been reported in the literature to date. We emphasize that a careful study considering gender and ethnic group makes procedures involving the region of lingula safer.

## 1. Introduction

The mandibular foramen (MF) corresponds to the opening of mandibular canal through which it penetrates the inferior alveolar vascular-nervous bundle [1], which is situated inferiorly and posteriorly to the greatest prominence of the lingula [2]. The lingula medially limits the MF [1]; thus, many authors consider this structure as an ideal anatomical landmark [3] to determine the position of the MF when performing certain surgical procedures, such as sagittal split ramus osteotomy (SSRO) technique [2]. In this procedure, the horizontal osteotomy should be made just above the lingula and extended posteriorly to it in order to make a safe split with less potential for nerve injury [4, 5].

The correct identification of MF is important to avoid complications not only during the performance of surgical procedures involving the region of lingula, but also in the inferior alveolar nerve anesthesia [6]. The authors report that this procedure may fail between 29% and 35% of cases and the anatomical variations are considered the major responsible for these failures [7, 8]. Inasmuch as this is an internal structure that cannot be palpated clinically, standard teaching is to use landmarks to estimate its location [9]. Many authors have used the lingula as a landmark for the location of the MF; however, they have not considered important variables such as the shape of lingula, gender, and race.

The purpose of this research was to study, in macerated adult human mandibles, the height of the lingula and provide morphometric data for its location considering aspects such as shape of the lingula, gender, and race. We intend to contribute to guide maxillofacial surgeons to perform a surgical procedure involving the region of lingula safer, preventing complications.

## 2. Material and Method

One hundred thirty-two macerated mandibles of Brazilian adult individuals, of both sexes, Amerindian and Caucasian, belonging to the Department of Morphology and Genetics, UNIFESP (São Paulo, Brazil), were used. Edentulous mandibles and/or those that did not contain information on sex, age, and race were excluded from this study. Only wholly or partially dentate hemimandibles that had at least one molar in the hemiarcade that are being studied were included.

**Figure 1:**
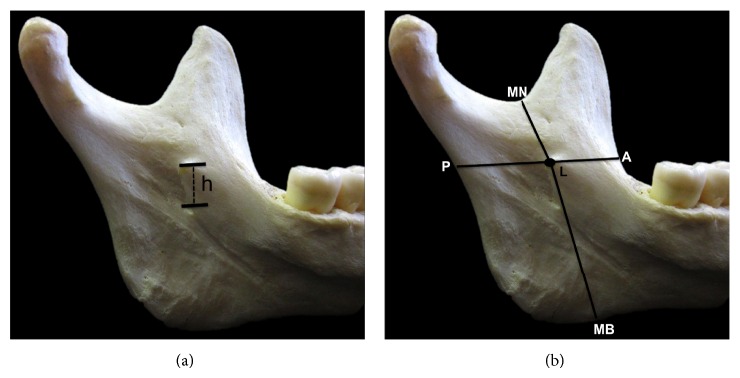
Medial view of the ramus of mandible. (a) Illustration of the height of the lingula (h). (b) Illustration of the distances measures: MN-L, A-L, P-L, and MB-L.

Regarding the shape, the lingula was classified into four types, according to Tuli et al. [10]: triangular, truncated, nodular, and assimilated. To determine the height of the lingula, the distance from the uppermost point of the lingula (L) to the lowest point of the mandibular foramen (MF) (L-MF) was mesured (Figure 1(a)). To determine the precise location of lingula, the following distances were mesured: from mandibular notch (MN) to lingula (MN-L); from anterior margin of ramus of mandible (A) to lingula (A-L); from posterior margin of ramus of mandible (P) to lingula (P-L); from mandibular base (MB) to lingula (MB-L) (Figure 1(b)).

To perform these measurements, we used a digital caliper. The variables such as gender and race were analyzed. The data obtained were tabulated and analyzed using ANOVA and* t student*, as applicable. Statistically significant *P* < 0.05 was considered.

Additionally we calculated the ratio of the lingula to obtain the anteroposterior position of lingula on the ramus of mandible, using the following formula: (A-L)/A-L + P-L. A smaller ratio of the lingula indicates that the lingula would be located at most anterior portion of ramus of mandible. To obtain the superoinferior position of lingula on the ramus of mandible, we used the following formula: MN-L + MB-L/3 to analyze whether it was located in the upper, medium, or lower thirds.

## 3. Results

The total was 253 hemimandibles, 96 Amerindian males (AM), 69 Caucasian males (CM), 62 Amerindian females (AF), and 26 Caucasian females (CF). In the statistical tests, we have not included the assimilated shape due to the small number of samples obtained.

### 3.1. Analysis Comparing Average Values Found for Each Type of Lingula in AM, CM, AF, and CF (Table 1)

Considering the height of the lingula, only in Amerindian males we found a statistically significant difference between truncated and nodular shape. The average values for males were similar in Amerindian and Caucasian individuals and higher than those found for females. Caucasian females showed average values lower than Amerindian females. Furthermore, the height for the nodular shape in Caucasian females was the lowest value found and the height for assimilated shape in Caucasian males was the highest value found.

Regarding the distance between lingula and mandibular notch (MN-L), we observed that the highest value found was for assimilated shape in CM and the lowest was for triangular shape in CF. In general, in all types of lingula in AM, the average values found were higher and in CM were lower.

With respect to distance from anterior margin of ramus of mandible to lingula (A-L), we observed a statistically significant difference for Amerindian males between truncated and triangular shapes, truncated and nodular shapes, and triangular and nodular shapes and for Amerindian females between truncated and nodular shapes, and truncated and triangular shapes. In truncated shape, we observed that the averages found for AM, CM, AF, and CF suffered no great variations, presenting themselves relatively similar; however, we observed change in this standard in triangular and nodular shapes, where CF exhibit much lower averages than those found for AM, AF, and CM. The highest average was found for AM in triangular shape and the lowest average was found for CF in nodular shape.

Regarding the distance from posterior margin of ramus of mandible to lingula (P-L), we observed that AM, AF, and CM showed similar averages and higher ones than those found for CF. The highest average was found for AM in nodular shape and the lowest average was found for CF in triangular shape.

Regarding the distance from mandibular base to lingula (MB-L), we have found that CM showed averages considerably higher than AM, AF, and CF in all types of lingula and CF showed the lowest values. The highest average was found for CM in truncated shape and the lowest was found for CF in nodular shape, being observed as a difference of 8.16 mm between these two averages.

### 3.2. The Lingula Ratio

Regarding the lingula ratio, we observed that the position of the lingula changes slightly according to the shape of lingula, sex, and race. Assimilated shape in Caucasian males was the type where the lingula occupied a most anterior portion of ramus of mandible and triangular shape in Amerindian males was the type where the lingula occupied a most posterior position.

In general terms, the nodular shape appeared to be occupied a most anterior portion of ramus of mandible and triangular shape most posterior in all individuals (Table 2).

### 3.3. Analysis of Superoinferior Position of the Lingula

For AM, the nodular and triangular shapes were located in the superior third of ramus of mandible and only the truncated shape was located in the middle third. For CM, all types were located in the superior third. For AF, only nodular shape was located in the superior third; the truncated and triangular shapes were located in the middle third. For CF, all shapes were located in the middle third.

We observed that in all cases the lingula was located near the intersection line between the superior and middle thirds, no wider than 1 mm, except in nodular shape for CF, which was in the middle third, 2 mm away from the intersection line between the superior and middle thirds.

### 3.4. Gender Analysis

Analyzing gender differences, we observed that there was a statistically significant difference for the truncated shape in the height of the lingula and in MN-L, P-L, and MB-L distances; for the triangular shape in MN-L, P-L, and MB-L distances, regarding the nodular shape, only A-L distance showed no difference between sexes (Table 3).

### 3.5. Analysis between Amerindian and Caucasian Individuals

Analyzing differences between Amerindian and Caucasian individuals, we observed that there was a statistically significant difference only in P-L distance for truncated and triangular shapes. Additionally, we found statistically significant difference between Amerindian and Caucasian males in P-L (*P* = 0.04) and MB-L (*P* = 0.0007). Between Amerindian and Caucasian females, we found statistically significant difference in A-L (*P* = 0.01), P-L (*P* = 0.005), and MB-L (*P* = 0.04) distances (Table 3).

## 4. Discussion

Morphometric data on the lingula and its location were studied in different researches, addressing different populations (Table 4); however, we found that there are no studies analyzing the location of the lingula according to the shape of the lingula in adult individuals; furthermore, we found no studies comparing races and there are few that analyze gender differences.

Sekerci et al. [11], studying a population of Turkish children, reported that there was statistical difference between gender in the triangular and nodular shapes. The height of lingula shows differences between genders and varies in different populations [12]. In our study, we found significant differences between males and females in height for truncated and nodular shapes. We observed that sexual differentiation was well set for other distances, such as MN-L, in the triangular and nodular shapes, P-L and MB-L for all shapes. Only for A-L distance, we did not observe difference between genders. Sekerci and Sisman [12] and Jansisyanont et al. [13] claim that, in females, the distances are shorter than or nearly equal to those found in males; we observed in our study that the mean values for height, MN-L, P-L, and MB-L were lower in females and only the A-L distance was lower or equivalent to those found in males, confirming that male mandibles are generally larger than female mandibles [9].

Amerindian females showed mean values higher than Caucasian females in height and in all distances, except in MN-L. Amerindian and Caucasian males showed similar values in height and in all distances, except MB-L in which Caucasian males showed higher average values. We found significant differences in P-L and MB-L between Amerindian and Caucasian males and in A-L, P-L, and MB-L between Amerindian and Caucasian females. According to our results, there was significant difference between Caucasian and Amerindian females in the anteroposterior location of the lingula; however, the distance from the lingula to the mandibular notch was quite similar.

Sekerci and Sisman [12] claim that the height of the lingula varies in different populations. In our study, in Brazilian adult mandibles, the height of the lingula was higher for CM in assimilated shape (9.38 mm) and lower for CF in nodular shape (6.54 mm). Lower values were found by Monnazzi et al. [2] (5.82 mm) and Samanta and Kharb [15] (5.5 mm), and higher values were reported in koreans (10.51 mm) [16]. The average values of the height of the lingula, which we found for males and females in our study, were similar to those reported by Sekerci and Sisman [12] in the Turkish population. Jansisyanont et al. [13] and Nicholson [18] found similar values to those that we found for males in our study. The mean values found for the height of the lingula, in our study, were determined according to the gender, race, and the lingula shape, having great variability. This data has great clinical relevance, since it can be used as a parameter to carry out certain surgical procedures involving the region of lingula.

In our study, the distance from the mandibular notch to the lingula (MN-L), distance that helps surgeons to find the lingula [12], was similar to the studies of Monnazzi et al. [2], Sekerci and Sisman [12], Jansisyanont et al. [13], Gite and Padhye [14], Viravudth and Plakornkul [19], and Kositbowornchai et al. [20]. Woo et al. [16] and Shah et al. [17] found values slightly higher than ours for Koreans and Indians, respectively. According to Sekerci and Sisman [12], the length of horizontal osteotomy in bilateral SSRO should be between 15 and 19 mm; according to Jansisyanont et al. [13], it should be between 17 and 24 mm. We disagree with these authors because in our study the distance from the mandibular notch to the lingula (MN-L) showed minimum values lower than 15 mm; furthermore, most authors report values lower than 17 mm [2, 5, 12–15]. Based on the results that were obtained through our study and on the literature data, we suggest that the length of horizontal osteotomy in bilateral SSRO should be between 13 mm and 24 mm. In our study, the differences between the average values for the MN-L distance are associated with ethnicity and gender. The average values that we found for females were lower than those that we found for males; moreover, the distances with higher average values were found for Amerindian males. We emphasize that a careful study considering gender and ethnic group makes procedures involving the region of lingula safer.

In our study, the distance from the anterior margin of the mandible to the lingula (A-L) with highest average value was found in triangular shape for Amerindian males (19.66 mm), and the lowest average value was found in assimilated shape for Caucasian males (12.77 mm). The average values found for females, in our study, were higher than those reported for the Turkish [12], while the average values found for males were similar. Higher mean values were found for North Indian and Thai, with 20 and 20.6 mm, respectively [12, 15]. In our study we observed great variability in the average values for A-L, with lower average values for assimilated and nodular shapes, allowing us to claim that the most anterior or posterior location of the lingula and consequently the location of the mandibular foramen are associated with each type of lingula. This distance showed no statistically significant difference between gender and race.

The distance from the posterior margin of the mandible to the lingula (P-L) was lower in the Turkish population [12] than that found in our study, in both females and males. Monnazzi et al. [2] found similar average values to those found in our study. The same occurred with Samanta and Kharb [15] who worked with North Indian population and with Woo et al. [16] who worked with Koreans. Jansisyanont et al. [13] reported the highest value found for this distance in the Thai population, with 18 mm. The lowest average value was found in our study for Caucasian female in triangular shape, with 11.17 mm. In our study, this distance showed marked sexual difference between races.

Regarding the distance from the mandibular base to the lingula (MB-L), the values found for the Turkish population [12] were similar to those found in our study. The values reported by Monnazzi et al. [2] were higher than ours. In our study, Caucasian males showed the highest mean values and Caucasian females the lowest. This distance showed marked difference between sexes.

The lingula ratio (LR) provides relevant data to aid in surgical planning. We note that, in general, lingulas with triangular shape are located slightly more posterior than lingulas with nodular shape, which can determine change in the level where the osteotomy should be performed in bilateral SSRO or the inferior alveolar nerve block, since the lingula marks the entrance of the inferior alveolar neurovascular bundle. The LR ranged from 0.47 for assimilated shape to 0.60 for triangular shape. The value reported in the literature for LR varied from 0.53 [13] to 0.56 [12, 15]. Based on our results, we agree with Kositbowornchai et al. [20] when they claim that the lingula was positioned slightly posterior to the center of the widht of the ramus.

As additional data for location of the lingula, we divided the ramus of mandible into three thirds: superior, middle, and inferior to analyze its superoinferior position. We observed that in all cases the lingula was located near the intersection line between the superior and middle thirds, except for the nodular shape in Caucasian females, who was in the middle third, 2 mm away from the intersection line, between the superior and middle thirds. The lingula was usually located in the middle third in females and in the superior third in males.

Based on our results, we emphasize that in planning a surgery involving the region of lingula, such as sagittal split ramus osteotomy (SSRO) technique, the surgeon must take into account aspects such as gender, race, and shape of lingula. Another important issue that should be considered is a possible anatomical variation in the medial region of the ramus of mandible, where there may be an accessory mandibular foramen located above the lingula [21, 22] or near the mandibular notch [23]. Such variations are not common but result in increased risk of complications involving the inferior alveolar nerve [21].

Alves and Cândido [24] affirm that the major factors involved in the failure of inferior alveolar nerve block are the accessory innervations and the improper placement of needle due to improper evaluation of landmarks. We consider it very important, when performing the inferior alveolar nerve block, to use the lingula as a landmark for the location of the MF considering aspects such as the shape of lingula, gender, and ethnic group. This is relevant not only for the success of anesthesia but also for preventing damage to vascular and neural elements.

## 5. Conclusions

This research provides additional data on height of the lingula and morphometric data for its location considering aspects such as shape of the lingula, gender, and race, information that had not been reported in the literature to date.

Comparing men and women, we found that the height of the lingula and the measured distances change according to the shape of the lingula (truncated, triangular, or nodular) determining variations in the lingula position. These changes were not so marked when comparing Amerindian and Caucasian individuals. Despite the gender, differences are more apparent than the differences between races; both of them must be taken into account in surgical procedures performed in the lingula region or inferior alveolar nerve block.

The mean values found for the height of the lingula, in our study, were determined according to the gender, race, and the lingula shape, showing great variability. This data has great clinical relevance, since it can be used as a parameter to carry out surgical procedures performed in the lingula region or inferior alveolar nerve block.

Considering the anteroposterior position, we conclude that the lingula was positioned slightly posterior to the center of the width of the ramus of mandible. Regarding the superoinferior position, the lingula was located in the superior or middle third of the ramus, in general, 1 mm away from the intersection line of these two thirds.

Based on our study and on the literature data, we suggest that the length of horizontal osteotomy in bilateral SSRO should be between 13 mm and 24 mm; however, we emphasize that a careful study considering gender and ethnic group makes procedures involving the region of lingula safer.

## Figures and Tables

**Table 1:** Average height of the lingula (in millimeters) and average of the following measurements: mandibular notch to lingula (MN-L); anterior margin of ramus of mandible to lingula (A-L); posterior margin of ramus of mandible to lingula (P-L); mandibular base to lingula (MB-L) (in millimeters), for Amerindian and Caucasian males and females, *P* value and standard deviation (SD).

	Amerindian male	Caucasian male	Amerindian female	Caucasian female	Total mean
Height					
Truncated	8.18	8.82	7.76	7.23	8.29
Triangular	8.41	8.44	7.47	6.76	SD ± 1.99
Nodular	9.33	9.19	8.01	6.54	
Assimilated	—	9.38	—	—	
Mean	8.50	8.89	7.78	7.00	
*P* value	0.04^*^	0.48	0.79	—	
SD	±1.79	±2.06	±1.87	±2.12	

MN-L					
Truncated	17.59	16.75	16.75	16.81	17.29
Triangular	18.36	17.4	16.26	14.96	SD ± 2.57
Nodular	17.95	17.50	16.12	16.38	
Assimilated	—	21.08	—	—	
Mean	17.90	17.35	16.53	16.43	
*P* value	0.45	0.56	0.95	—	
SD	±2.70	±2.49	±2.50	±1.88	

A-L					
Truncated	18.00	17.8	18.39^*^	17.34	17.76
Triangular	19.66	18.35	18.59^*^	16.69	SD ± 2.69
Nodular	16.52	16.98	16.39^*^	13.79	
Assimilated	—	12.77	—	—	
Mean	18.18	17.47	17.97	16.57	
*P* value	<0.0001^*^	0.29	0.005^*^	—	
SD	±2.46	±2.96	±2.40	±2.88	

P-L					
Truncated	16.10	15.75	14.76	13.63	15.28
Triangular	15.68	14.44	14.04	11.17	SD ± 2.31
Nodular	16.80	15.86	14.83	12.86	
Assimilated	—	14.17	—	—	
Mean	16.13	15.41	14.67	13.22	
*P* value	0.23	0.05	0.56	—	
SD	±2.27	±2.10	±2.13	±1.85	

MB-L					
Truncated	32.90	36.16	32.58	30.77	33.30
Triangular	34.80	35.80	31.38	29	SD ± 4.14
Nodular	32.87	35.03	30.68	28	
Assimilated	—	31.01	—	—	
Mean	33.47	35.46	31.97	30.16	
*P* value	0.07	0.51	0.33	—	
SD	±3.79	±3.48	±4.72	±2.57	

(—) could not be calculated; ^*^statistically significant difference. The *P* value was found on analysis of the shapes of lingula in each group studied.

**Table 2:** Lingula ratio found for each shape of lingula in Amerindian male, Caucasian male, Amerindian female, and Caucasian female.

	Amerindian male	Caucasian male	Amerindian female	Caucasian female
Truncated	0.52	0.53	0.55	0.55
Triangular	0.60	0.55	0.56	0.59
Nodular	0.49	0.51	0.52	0.51
Assimilated	—	0.47	—	—

(—) could not be calculated.

**Table 3:** *P* value (between male and female, Amerindian and Caucasian individuals) for height and the following measurements: mandibular notch to lingula (MN-L); anterior margin of ramus of mandible to lingula (A-L); posterior margin of ramus of mandible to lingula (P-L); mandibular base to lingula (MB-L).

	Truncated		Triangular		Nodular	
	M × F	C × A	M × F	C × A	M × F	C × A
Height	0.026^*^	0.91	0.136	0.46	0.0001^*^	0.88
MN-L	0.199	0.21	0.030^*^	0.99	0.021^*^	0.811
A-L	0.787	0.27	0.765	0.32	0.151	0.064
P-L	<0.0001^*^	0.03^*^	0.004^*^	0.04^*^	0.010^*^	0.432
MB-L	0.004^*^	0.09	0.0008^*^	0.46	0.001^*^	0.055

^*^Statistically significant. Male: M; female: F; Caucasian: C; Amerindian: A.

**Table 4:** Mean values (in millimeters) for height and the following measurements: mandibular notch to lingula (MN-L); anterior margin of ramus of mandible to lingula (A-L); posterior margin of ramus of mandible to lingula (P-L); mandibular base to lingula (MB-L) reported by other authors.

Authors	References	Population	Height	MN-L	A-L	P-L	MB-L
Monnazzi et al.	[2]	—	5.82	16.38	16.5	14.63	27.09 —

Kim et al.	[5]	Koreans	—	15.1	17.4	—	—

Sekerci and Sisman	[12]	Turkish	7^RF^ 9.08^RM^ 7.24^LF^ 8.58^LM^ 7.97^*^	13.95^RF^ 17.21^RM^ 14.17^LF^ 15.93^LM^ 15.3^*^	15.97^RF^ 18.23^RM^ 15.6^LF^ 17.26^LM^ 16.7^*^	12.43^RF^ 13.61^RM^ 12^LF^ 14.03^LM^ 13^*^	30.93^RF^ 35.53^RM^ 30.94^LF^ 36.32^LM^ —

Jansisyanont et al.	[13]	Thai	8^RM^ 8.1^RF^ 8.4^LM^ 8.5^LF^ 8.2^*^	16.9^RM^ 16^RF^ 16.8^LM^ 15.9^LF^ 16.6^*^	20.9^RM^ 20.2^RF^ 20.6^LM^ 20.1^LF^ 20.6^*^	18.2^RM^ 18.4^RF^ 17.4^LM^ 17.3^LF^ 18^*^	— — — —

Gite and Padhye	[14]	Indian	—	16.2	—	—	—

Samanta and Kharb	[15]	North Indian	5.5	15.4	20	15	—

Woo et al.	[16]	Koreans	10.51	19.82	18.6	16.1	—

Shah et al.	[17]	Indian	— —	18.19^L^ 18.28^R^	— —	— —	— —

Nicholson	[18]	East Indian	8.6^R^ 9.1^L^	— —	— —	— —	— —

Viravudth and Plakornkul	[19]	Thai	8.7^R^ 8.2^L^	— —	— —	— —	— —

Unreported or with different measurement: (—); left: L; right: R; male: M; female: F; mean: ∗.
